# Entropy in Image Analysis II

**DOI:** 10.3390/e22080898

**Published:** 2020-08-15

**Authors:** Amelia Carolina Sparavigna

**Affiliations:** Department of Applied Science and Technology, Polytechnic University of Turin, 10129 Turin, Italy; amelia.sparavigna@polito.it

**Keywords:** image entropy, image processing, image encryption, medical imaging, neural engineering, computer vision, crowd motion detection, security

Image analysis is a fundamental task for any application where extracting information from images is required. The analysis needs numerical and analytical methods that are highly sophisticated, particularly for those applications in medicine, security, and other fields where the results of the processing consist of data of vital importance. This fact is evident from all the articles composing this Special Issue of Entropy in which authors have widely tested methods to verify their results. On the other hand, being specifically involved in numerous applications, image analysis is producing a large number of approaches and related algorithms in which the variety is clearly exemplified by the case studies proposed in this issue. Let us stress that, in its progression, an important stimulus and cross-fertilization among publications was observed with the editor’s great pleasure.

Let us describe the articles of the issue shortly. 

In Reference [1], we can find a problem of medical imaging based on the ultrasound addressed. It is the analysis of the severity of Duchenne Muscular Dystrophy. Ultrasound imaging enables routine examinations for which entropy represents a great help in visualizing related changes. Using small-window entropy, the imaging technique exhibits higher diagnostic performance than conventional methods. Article [2] is also considering ultrasound imaging. The authors’ aim was that of discriminating the normal muscles from neuropathic muscles in children affected by Pompe disease. The method is using a texture-feature parametric imaging that simultaneously considers microstructure and macrostructure. In Reference [3], a compression method is proposed, which can be very useful in telemedicine applications. The addressed and solved problem is that of having a lossless compression of images of malaria-infected red blood cells. In fact, a remote diagnosis of malaria infection could receive a great benefit from efficient compression of high-resolution images.

In Reference [4], we are again in the field of medical imaging. In the article, a blind image quality assessment (BIQA) method for evaluating magnetic resonance images is introduced. Images are first preprocessed to reach acceptable local intensity differences. Quality is expressed by the entropy coming from a thresholding in sequence. Image Quality Assessment appears in Reference [5] as well. The authors are approaching the problem of training IQA, using deep neural networks. Since the image quality is highly sensitive to changes in entropy, a new data expansion method based on this remarkable quantity is proposed.

A security scheme for medical imaging is the subject of article [6], which is presenting a medical image stego-hiding scheme, named BOOST by the authors. It uses a pseudorandom byte output technique based on the nuclear spin generator. The security analyses show that BOOST can be used for secure medical record communication. An image encryption scheme appears in Reference [7] as well. The scheme is based on quasi-resonant Rossby/drift wave triads and Mordell elliptic curves. A dynamic substitution box is employed for the plain image. The security of the proposed scheme was tested and compared with other popular schemes. Article [8] proposes an algorithm for medical color images encryption. It uses chaotic systems to protect medical images against attacks. The algorithm has two main parts: a high-speed permutation process and an adaptive diffusion. Entropy obtained after experiments tells that the algorithm is suitable for this type of image. 

Chaos and hyper-chaos, combined with DNA coding, appears in an image encryption algorithm proposed in Reference [9]. The first stage involves three rounds of scrambling. Then a diffusion algorithm is applied to the plaintext image, and the intermediate ciphertext image is partitioned. The final encrypted image is formed by using DNA operation. Additionally, in Reference [10], we find an image encryption based on a hyperchaotic system proposed with a pixel-level filtering obtained by means of variable kernels. A global bit-level scrambling is also conducted to change the values and positions of pixels simultaneously. At the end of the process, a DNA-level diffusion is used as well. 

Another security problem and related analysis of an image chaotic encryption algorithm is given in Reference [11]. The proposed algorithm generates Latin-bit cubes and uses them for image chaotic encryption. The algorithm also uses different Latin cube combinations to scramble the diffusion image. In Reference [12], the encryption scheme is based on double chaotic S-boxes. A compound chaotic system, Sine-Tent map, is proposed to widen the chaotic range and improve the chaotic performance. Data hiding is another very crucial research topic in information security [13]. In this article, the authors are proposing a high-capacity data-hiding scheme for absolute moment block truncation coding (AMBTC) decompressed images. For the secret data string, a unique encoding and decoding dictionary is involved, and it is used in embedding and extraction stages.

The issue of image retrieval based on a convolutional neural network (CNN) has been considered by the authors of Reference [14]. The article is proposing a feature distribution entropy to measure the difference of regional distribution information in the feature maps from CNNs. Experiments have been conducted on public datasets. Another application of entropy is available in Reference [15] to improve the methods of image binarization for automatic text recognition in images acquired in uncontrolled lighting conditions. The preprocessing of images is made by means of a local entropy filtering. 

Computer vision is the subject of Reference [16]. The article is proposing a novel network structure, which is involving a pipeline guidance strategy for the detection of human key-points. The use of a pipelined guidance allows one to find a balance between the convolution calculations and the communication time in order to improve the training speed of the network. In addition to the computer vision in the issue, we can find neural engineering research. In Reference [17], an application of the continuous wavelet transform and convolutional neural network for brain-computer interface is proposed. The article includes a novel motor imagery classification scheme with the aim of capturing highly informative electroencephalogram images. 

Two forms of normalized entropy are used in Reference [18] for evaluating the evolution of neuro-aesthetic variables, displayed by portrait paintings, from Early Renaissance to Mannerism. The variables included symmetry, balance, and contrast (chiaroscuro) as well as intensity and spatial complexities. In Reference [19], the application is an image-based denomination recognition of Pakistani currency notes. The authors propose a procedure in two steps that extracts a currency note from the image background via local entropy and range filters. Then, the aspect ratio of the extracted currency note is calculated to determine its denomination. In Reference [20], a methodology based on weld segmentation and entropy is proposed such as an evaluation by conventional and convolution neural networks to assess the quality of welds. Compared to conventional neural networks, the method does not require image preprocessing. The performed experiments show that the best results are achieved using convolution neural networks. The image evaluation for engine flame is the subject of the investigation proposed in article [21]. In it, we find the detectability of the related infrared radiation. The influence of the earth background interference on plume radiation detection is investigated and discussed in detail. 

Let us conclude with an application that can be very useful for controlling the movements of a crowd. In Reference [22], we find an article proposing a method for salient crowd motion detection based on direction entropy and a repulsive force network. This work focuses on the manner by which it is possible to detect the salient regions effectively. Let us observe that the proposed method could be integrated in any crowd check-point, such as during a pandemic, for helping in the control of disease spread. 
